# 896 Trauma-Burn Induced Dermatologic Lesions Following Split Thickness Skin Graft: A Case Report and Literature Review

**DOI:** 10.1093/jbcr/iraf019.427

**Published:** 2025-04-01

**Authors:** Rebeka Dejenie, Ian Powelson

**Affiliations:** University of California Davis; University of California Davis

## Abstract

**Introduction:**

Eruptive keratoacanthoma (EKA) development at a split-thickness skin graft donor site is rare and medically challenging. We present a case of a 47-year-old man with 8% TBSA burns to the left lower extremity. He received split-thickness skin grafts from his right thigh and had an uneventful postoperative recovery. However, seven weeks later, a non-painful nodular lesion appeared at the donor site, followed by an eruption of multiple similar lesions. A biopsy confirmed EKA. Though rare, EKA and other dermatologic complications can arise at graft donor sites. Given the widespread use of split-thickness skin grafts in burn care, providers must be aware of potential skin complications at these sites. This study aimed to review the existing literature to identify trauma-induced dermatologic conditions occurring within split-thickness skin graft donor sites.

**Methods:**

First, a search was conducted to explore dermatologic complications associated with trauma. A literature review was then performed using PubMed, Google Scholar, and Medline, specifically examining dermatologic issues at donor sites following split-thickness skin grafts.

**Results:**

The review identified four rare dermatologic conditions that developed in split-thickness graft donor sites across 22 articles: herpes zoster, SCC, keratoacanthoma, and bullae. Of the articles, 1 (4.5%) studied herpes zoster, 12 (54.5%) focused on SCC, 4 (18.1%) examined keratoacanthoma, and 5 (22.7%) reviewed bullae. Our findings indicate that herpes zoster eruption in a donor site, although rare, can lead to fatal results for patients. This highlights the need for awareness of burn-related immunosuppression and its potential consequences, including disseminated herpes zoster infection. SCC and keratoacanthoma also demand rapid and accurate diagnosis due to their differing management yet similar presentation. While SCC often requires excision to prevent progression of disease, keratoacanthoma calls for different management which vary from surgery to surveillance. Lastly, bullae, although rare, has increased in incidence in the past two decades, requiring distinct treatments ranging from steroids to immunosuppressive therapies. Each of these conditions can significantly impact patient outcomes and necessitate vigilant surveillance of graft donor sites.

**Conclusions:**

Split thickness skin grafts can lead to trauma-induced dermatologic conditions, including squamous cell carcinoma, keratoacanthoma, herpes zoster, and bullae. Given the immunocompromised status of burn patients, routine surveillance of both graft donor and recipient sites is crucial. Accurate diagnosis of these conditions are crucial for burn providers to ensure timely and appropriate management.

**Applicability of Research to Practice:**

This case underscores the importance for burn care providers to recognize, diagnose, and manage postoperative trauma induced dermatologic complications in graft donor sites.

**Funding for the Study:**

N/A

